# Widespread Existence of Quorum Sensing Inhibitors in Marine Bacteria: Potential Drugs to Combat Pathogens with Novel Strategies

**DOI:** 10.3390/md17050275

**Published:** 2019-05-08

**Authors:** Jing Zhao, Xinyun Li, Xiyan Hou, Chunshan Quan, Ming Chen

**Affiliations:** 1 Key Laboratory of Biotechnology and Bioresources Utilization (Dalian Minzu University), Ministry of Education, Dalian 116600, China; zhaojing@dlnu.edu.cn (J.Z.); lixinyun9118@163.com (X.L.); xyhous@dlnu.edu.cn (X.H.); 2 College of Life Science, Dalian Minzu University, Dalian 116600, China; 3 School of Biological Engineering, Dalian Polytechnic University, Dalian 116600, China

**Keywords:** marine bacteria, QS inhibitors, small molecule QS inhibitors, quorum quenching enzymes, QS inhibition mechanisms, structural modification, application

## Abstract

Quorum sensing (QS) is a phenomenon of intercellular communication discovered mainly in bacteria. A QS system consisting of QS signal molecules and regulatory protein components could control physiological behaviors and virulence gene expression of bacterial pathogens. Therefore, QS inhibition could be a novel strategy to combat pathogens and related diseases. QS inhibitors (QSIs), mainly categorized into small chemical molecules and quorum quenching enzymes, could be extracted from diverse sources in marine environment and terrestrial environment. With the focus on the exploitation of marine resources in recent years, more and more QSIs from the marine environment have been investigated. In this article, we present a comprehensive review of QSIs from marine bacteria. Firstly, screening work of marine bacteria with potential QSIs was concluded and these marine bacteria were classified. Afterwards, two categories of marine bacteria-derived QSIs were summarized from the aspects of sources, structures, QS inhibition mechanisms, environmental tolerance, effects/applications, etc. Next, structural modification of natural small molecule QSIs for future drug development was discussed. Finally, potential applications of QSIs from marine bacteria in human healthcare, aquaculture, crop cultivation, etc. were elucidated, indicating promising and extensive application perspectives of QS disruption as a novel antimicrobial strategy.

## 1. Introduction

Quorum sensing (QS) has been recognized as a widespread phenomenon in bacteria. To date, QS systems have been elucidated in detail in many different bacterial species, especially in Vibrio. In QS systems, signal molecules are secreted by bacteria in a cell density-dependent manner and induce signal transduction through cascaded QS regulatory proteins [1,2]. Signal molecule-mediated QS would participate in regulation of multiple phenotypes and important physiological functions, including cell metabolism, release of virulence factors, stress response, etc., in bacteria [3,4]. 

Recently, QS-mediated virulence expression, biofilm formation, and colonization of pathogenic bacteria have aroused great concern [5,6,7]. Due to the multidrug resistance of certain pathogens, QS interruption or inhibition seemed to be a promising strategy to combat pathogen infection [8,9]. QS interruption can be performed through mainly three mechanisms: blocking the synthesis of signaling molecules, degradation of signaling molecules, and impeding the binding of signaling molecules to receptor proteins in QS pathways [10,11]. Theoretically, QS interruption can be realized through different methods based on above mechanisms. However, certain methods are not feasible, e.g., reconstruction of bacterial metabolic pathways to inhibit the synthesis of signal molecules or changing environmental conditions (pH and temperature) for chemical degradation of signal molecules. QS inhibitors (QSIs), due to their feasibility and applicability in combating pathogens, are widely studied in recent years. 

QSIs mainly include two types: one type is small molecule QSIs either extracted from natural resources or obtained by chemical synthesis [12,13], and the other type is quorum quenching enzymes, mainly including acylases, lactonases targeting acyl-homoserine lactones (AHLs) as signaling molecules, AI-2 kinases targeting furanosyl borate ester (autoinducer 2, AI-2) as signaling molecules, etc. [14,15,16]. Natural QSIs have attracted great attention due to three reasons: firstly, natural resources have enormous potential to be tapped; secondly, structural modification based on natural QSIs would likely to provide novel QSIs with higher efficiency; and thirdly, natural QSIs are expected to be environmentally compatible and safe, which is beneficial for novel drug development and environment governance. Natural QSIs have been identified in both prokaryotic and eukaryotic origin, which include both terrestrial animals, plants, microorganisms and marine organisms [12,17,18,19,20]. To date, QSIs or organisms with QS inhibition capability have been found in vast marine resources. Unlike quorum quenching enzymes which were discovered mainly from marine bacteria extracts, small molecule QSIs originated from more diversified sources, including marine animals, algae, sponge, coral, fungi, and bacteria in the broad sense [21,22,23,24,25,26]. Also, certain marine-derived microorganisms were discovered with QS inhibition activity without identification of QS inhibitory substances [27].

To date, many small molecule QSIs from marine microorganisms have been mentioned and researched [28]. In this article, we made a systematical elaboration of marine bacteria with QS inhibition capabilities and their production of QSIs. We first summarized the screening of marine bacteria with QS inhibition capabilities. Afterwards, two types of marine bacteria-derived QSIs—small molecule QSIs and quorum quenching enzymes—were elucidated respectively from the aspects of sources, structures, QS inhibition mechanisms, environmental tolerance, effects/applications, etc. Structural modification of small molecule QSIs for higher efficiency was further focused for future drug development. Finally, QS inhibition mechanisms and potential applications of the marine bacteria-derived inhibitors were elucidated.

## 2. Screening and Classification of Marine Bacteria with QS Inhibition Capabilities

To discover QSIs in the vast marine bacteria sources, many researchers have used different screening methods or systems for prescreening or large-scale screening of marine bacteria with potential QS inhibition capabilities. The commonly used screening systems are based on phenotype changes of biosensor reporter strains, including bioluminescence of *Vibrio harveyi* BB120, *Escherichia coli* pSB1075, *Pseudomonas aeruginosa* PAO-JP2, pigment production of *Serratia marcescens* SP15, *Serratia rubidaea* JCM 14263, *Chromobacterium violaceum* CV026 and DSM 30191, VIR07, β-galactosidase activity of *Agrobacterium tumefaciens* A136, KYC55, NTL4, etc. Screening based on the biosensor strains is a simple and high-throughput method for exploring marine bacteria with QS inhibition activity. Besides the biosensor strains, metagenomic sequencing was also used for rapid and large screening of QS-inhibitory bacteria in recent years, which can unveil the frequency of quorum quenching enzyme sequences in marine bacteria [29]. This technique avoids the defects of biosensor reporter strains, which could only detect the QS inhibition activity of cultivable bacteria. Also, marine metagenomic sequencing provides a comprehensive search for putative quorum quenching enzymes, thus providing a vast reservoir of marine-derived quorum quenching enzymes for research and utilization. 

**Figure 1:**
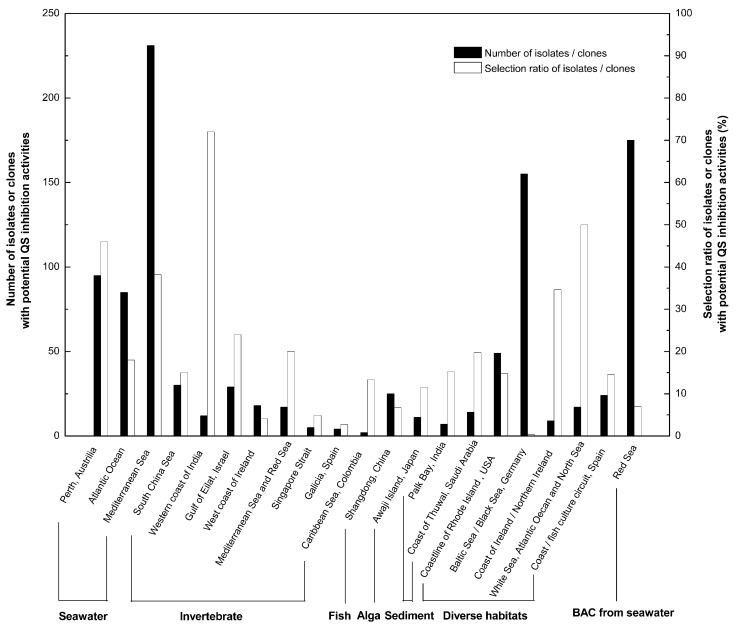
Large-scale prescreening showed abundance of marine bacteria with potential quorum sensing (QS) inhibition activities.

Screening from various marine environments using either biosensor strains or metagenomic sequencing showed abundance of QS-inhibitory marine bacteria (Figure 1) [30,31,32,33,34,35,36,37,38,39,40,41,42,43,44,45,46,47,48,49]. It could be seen that QS-inhibitory marine bacteria were mainly screened out from sea waters, marine sediments, as well as marine invertebrates, fish, algae, etc. These origins scattered in different regions and cities in the world. 

In large-scale screening of QS-inhibitory bacteria, three interesting phenomena were found. First, marine bacteria might not only have the ability to interfere with AHL-mediated QS, but also have the ability to interfering with AI-2/QS systems [39,45], indicating a wide application of QS-inhibitory marine bacteria against pathogens with both AHL and AI-2 mediated QS systems. Another notable point was that the depth of sea water might positively correlate with the quantity of QS-inhibitory marine bacteria discovered. This discovery might guide us to explore deep sea microorganisms for QS inhibitory substances. Thirdly, it is interesting to notice that pathogens associated with marine eukaryotes also have QS-inhibitory activities, which might help pathogens compete for adhesion with other bacteria that foul the surfaces of marine eukaryotes with biofilm formation [34,45]. The living of pathogens via QS-interfering is worth studying for future prevention of certain marine bacterial diseases. Of course, prescreening results might not be quite accurate and false positive results might always exist, since different biosensor reporter strains and different culture media for screening might vary in effectiveness for bacteria isolation [34,35,38].

**Figure 2:**
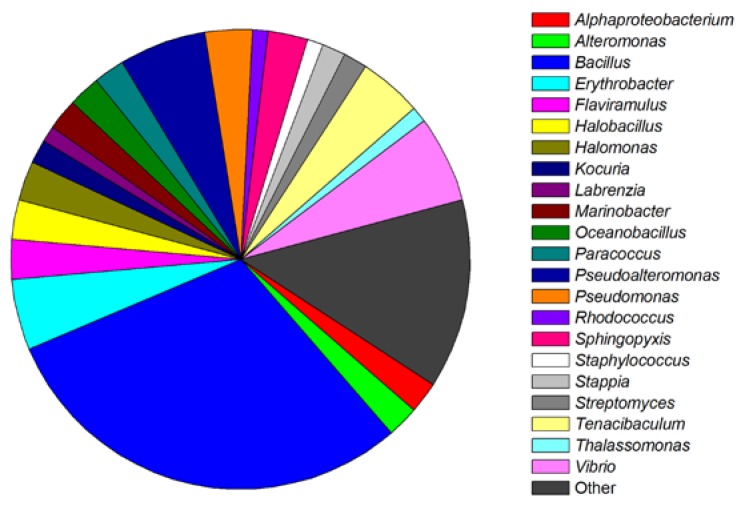
Classification and relative abundance of the marine bacteria isolates with potential QS inhibition activities. The genera represented by a single isolate are grouped as “other”.

Based on screening, many researches have isolated one or several QS-inhibitory bacteria strains from marine origins. The identified bacteria, which have potential QS inhibition ability but have not been further explored for specific QSIs, were categorized in Figure 2 [33,34,35,37,38,40,41,42,43,44,46,47,48,49,50,51,52,53,54,55]. Statistically, QS-inhibitory bacteria could be divided into four phylums and five classes. The phylums include Proteobacteria (47.22%), Firmicutes (37.78%), Bacteroidetes (8.89%), and Actinobacteria (6.11%). The five classes include Alphaproteobacteria (20.56%), Gammaproteobacteria (26.67%), Actinobacteria (6.11%), Bacilli (37.78%), and Flavobacteria (8.89%).

Besides many QS-inhibitory bacteria that have been identified, certain QS-inhibitory marine bacteria cultures remained to be disclosed. Tinh et al. isolated AHL-degrading bacterial enrichment cultures from the digestive tract of Pacific white shrimps. One of the enrichment cultures could improve turbot larvae survival, possibly through a QS-interference strategy. However, since the enrichment cultures contained a variety of bacteria, the species with actual AHL-degrading ability remained to be identified [56,57]. Cam et al. also isolated AHL-degrading bacterial enrichment cultures from the gut of European Seabass in Belgium and Asian Seabass in Vietnam, which could improve prawn larvae survival [58]. Also, the enrichment culture remained to be studied further.

The vast resource of QS-inhibitory marine bacteria needs to be further explored. To confirm the QS-inhibitory activity of bacteria, purification of the active substance, high-performance liquid chromatography–mass spectrometry (HPLC-MS) analysis and usage of other techniques should be carried out, either to identify the chemical structures of small molecule QSIs or to detect the degradation of QS signal molecules by quorum quenching enzymes.

## 3. Small Molecule QSIs Derived from Marine Bacteria

### 3.1. Natural Small Molecule QSIs

To date, natural small molecule QSIs of different structures and origins have been isolated and identified from a variety of marine bacteria. Structures, origins, anti-QS working concentrations, mechanisms, and effects of the small molecule QSIs are outlined in Table 1. Based on structural characteristics, small molecule QSIs could be divided into five categories, including cyclic and linear peptides, amides, fatty acids and phenol derivatives, AHL analogs, and others. From the aspects of anti-QS mechanisms, the reported QSIs might antagonize AHL, (*S*)-3-hydroxytridecan-4-one (CAI-1) and AIP signal molecule-mediated QS systems both in Gram-negative and Gram-positive bacteria. The working mechanisms of reported small molecule QSIs could be divided into mainly four types, including competition for receptor proteins with signal molecules, interfering in the stability of receptors, blocking the expression of signal molecule receptor protein, as well as inhibiting signal molecule synthesis by binding to AHL synthase. Certain types were newly proposed modes of action for QS inhibition and remained to be verified. It is noticeable that certain marine bacteria-derived chemical compounds might function as precursors of QSIs, such as polyhydroxy butyrates (PHB), which might be degraded by PHB depolymerase in the pathogen Vibrio. Although PHB addition in the Vibrio culture could inhibit QS, due to the insolubility of PHB in water, the degradation product β-hydroxy butyric acid might actually function as the QSI [68]. 

It is interesting to notice that certain suspected QSIs shared structural or functional similarities with different types of signal molecules, e.g., AHLs, AI-2 and autoinducing peptides (AIPs). For AHLs analogs, Bruns et al. mentioned *N*-Acylated amino acid methyl esters produced by marine Roseobacter group bacteria isolated from macroalgae. These compounds had structural similarities with QS signaling molecule AHLs and have antagonistic activity against other microorganisms in vicinity [77]. Phenethylamides were also indicated as AHL structural mimics and might function as QSIs by competing for receptor binding [66]. Also, certain probiotic bacteria were reported to produce AHLs which could possibly compete with different structural AHLs in other pathogens, thus inhibiting their QS regulation pathways and virulence [73]. A suspected QSI which might work as AI-2 signal molecules was also discovered. Pentadecanal, a long-chain fatty aldehyde from Antarctic marine bacterium *Pseudoalteromonas haloplanktis*, could suppress biofilm formation of *Staphylococcus epidermidis*, possibly by interference with the AI-2/QS system [78]. For AIP analogs, ngercheumicins and solonamides were found as potential QSIs sharing similar structural traits with the AIPs of *S. aureus* [62,63]. Besides the above mentioned suspected QSIs, diketopiperazines (DKPs) were also proposed as potential QSIs [53], which were previously found to serve as QS signal molecules in *Burkholderia* sp. These signal molecule analogs, due to their structural features or functions, might have dual functions as both QSIs and QS signal molecules. It is possible that they serve as QS signal molecules either for their producing bacteria or for other bacteria in the same community, exerting cross-species QS manipulation.

Besides above-mentioned QSIs, certain researches also isolated some suspected QSIs, of which the chemical structures or anti-QS activities were not determined or verified. Durai et al. found that marine sponge associated bacteria *Alcaligenes faecalis* contained an active fraction with anti-QS activity. However, the isolated substance was not yet determined as QSIs exactly [79]. Also, Clark et al. isolated several tumonoic acids from a marine cyanobacterium as possible QSIs, which showed only modest QS inhibition activities with the assay of one biosensor strain but no QS inhibition activities with another biosensor strain [80]. Ibacache-Quiroga et al. reported a biosurfactant as a potential QS inhibitor, which was produced by a marine bacterium *Cobetia* sp. isolated from seawater samples in intertidal coastal ponds in Chile. The biosurfactant was suspected to be a mixture of 3-hydroxy fatty acids, which could interfere with QS of a fish pathogen and repress its QS-controlled virulence gene expression. The interfering mechanism might be that the biosurfactant and AHLs form lipid aggregates [81]. In summary, natural small molecule QSIs remained to be isolated, purified, and researched for their anti-QS activities and mechanisms. To further disclose and elucidate anti-QS mechanisms, multiple techniques should be used, especially molecular biology and structural biology techniques, e.g., combination of molecular docking methods and dynamics simulations, as well as crystal structure analysis of the complex of QSI, signal molecule receptor, etc.

### 3.2. Structural Modification of Natural Small Molecule QSIs

Researches of natural QSIs are now faced with two problems: Firstly, extraction from marine bacteria yielded only small amounts of QSIs due to the low content of natural QSIs in marine bacterial metabolites, the loss in complicated purification procedures and the instability of the natural QSIs; Secondly, certain natural QSIs were not quite effective, even toxic to target bacteria, which greatly limited their potential applications. To solve above two problems, chemical synthesis and structural modification of natural QSIs might be an alternative choice. 

In early and recent studies focusing on structural modification of QSIs from marine origins, one common target was natural furanones isolated from macroalga. Since furanones might be toxic to aquatic organisms, synthetic QSIs based on their structures have been designed and tested. A synthetic furanone derivative C-30 has been proven to enhance QS-inhibitory activity and could effectively inhibit virulence gene expression in *P. aeruginosa* [82]. Lactam analogs of the QS inhibitor fimbrolides—a class of halogenated furanones isolated from the red marine alga—were synthesized and proved to have QS-inhibitory activities [83]. Brominated thiophenones, sulfur analogs of furanones, were also proved as effective QSIs, however, they were found to be toxic to the tested aquatic organisms [84].

**Figure 3:**
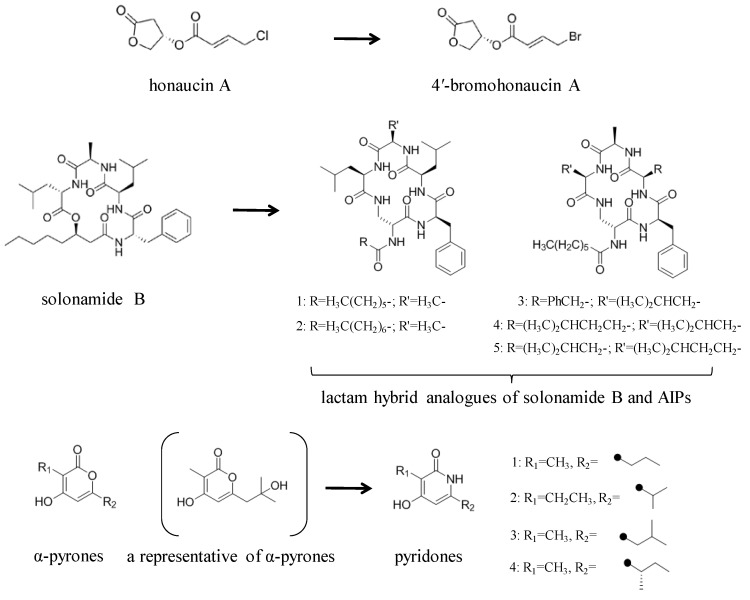
Structure modification of natural marine bacteria-derived QSIs/chemical compounds to obtain more potent QSIs. To the left of the arrow are natural QSIs or natural chemical compounds without QS-inhibitory activity. To the right of the arrow are novel QSIs after structure modification.

Structural modification of QSIs from marine bacteria was rarely mentioned only until recent years. Choi et al. has synthesized honaucin A (a natural QS inhibitor isolated from a marine cyanobacterium) and several derivatives using the fragment-based drug design strategy. The derivatives were designed based on alcohol unit, acid unit, and the whole structure of honaucin A. Evaluation proved that halogen substitution could effectively enhance the QS-inhibitory activity, which was dozens of times higher than that of honaucin A. Among the 23 candidates, 4′-bromohonaucin A seemed to be the most promising QSI [74]. Hansen et al. designed a series of lactam hybrid analogs of solonamide B (from marine *Photobacterium halotolerans*) and AIPs, such as *S. aureus* AgrC QSIs. The structural modification targeted expansion of ring size, type of side chain, amino acid substitutions and stereochemistry. The key factors in structures necessary for QS-inhibitory activity include the presence of a short fatty acid tail, the optimal tail length, all-D stereochemistry, Phe residue in the 2-position and Leu in 5-position, etc. Out of 27 analogs, five most efficient were screened out, the QS-inhibitory activities of which were also dozens of times higher than the compound before structural optimization [85]. Kwan et al. also compared the QS-inhibitory activity of lyngbyoic acid (from a marine cyanobacterium) with its structural related compounds. It was indicated that the fused cyclopropane “tag” in lyngbyoic acid, the free acid moiety, and longer alkyl chains might be crucial for the QS-inhibitory function [69]. Du et al. isolated several α-pyrones (from a *Streptomyces* sp. derived from marine algae) without QS-inhibitory activities. However, several―α-pyridones (pyridin-2(1H)-ones) obtained by the diversity enhanced extract method―showed QS-inhibitory activities [86]. This indicated that marine-derived natural compounds could also be considered as precursors of potential QSIs. The structures of above mentioned natural QSIs and their most promising derivatives were summarized in Figure 3. 

The abundance of QS-inhibitory bacteria and natural compounds in marine environments provides us with great opportunities to create more novel and efficient QS-inhibitory compounds based on the identified natural structures. In structural modification, we should fully concern structure–activity relationships. Once the essential structural features responsible for QS-inhibitory activity are revealed, analogs of natural QSIs with high potency would be designed and synthesized more rationally. Since certain QSIs resemble the structures of signal molecules of different QS systems, as mentioned above in Section 3.1, a novel QS inhibitor design could also be performed based on the natural structures of AHLs, AI-2, AIPs, etc. Small alterations in the signal molecule structural motifs might lead to effective QS antagonists. To test the QS-inhibitory activities of the structure-modified compounds, traditional assay with QS biosensor strains and computer-aided molecular docking with the crystal structures of signal molecule receptors should be used in combination. The latter method could provide a high-throughput screening for effective QSIs. It could also help researchers to identify critical functional groups in the modified QSIs and explain their anti-QS mechanisms.

Besides chemical synthesis and structural modification, biosynthesis of QSIs might be another consideration. Study of the whole-genome sequence of the strains producing QSIs might provide insights for the clear biosynthetic routes of the inhibitors, thus facilitating more effective biosynthesis. 

## 4. Quorum Quenching Enzymes Derived from Marine Bacteria

Quorum quenching activities have been reported widely in marine bacteria. For example, Romero et al. found that 85 out of 464 strains isolated from seawater could eliminate AHLs (C12-HSL), in which two strains with wide substrate spectrum were deduced to have lactonase activity. Also, a relatively high frequency of quorum quenching genes in marine metagenomes was discovered by blast [29]. Muras et al. also found a high prevalence of quorum quenching enzyme sequences in metagenomic samples in the Mediterranean Sea. They found that the relative abundance of acylase sequences increased in the deep sea while lactonase sequences seemed to distribute more averagely in different depth of seawater [38].

To date, although quorum quenching enzymes have been widely reported, these types of enzymes of marine origin have not been fully revealed in gene sequences and characteristics. Many studies only discovered marine bacteria with AHL degrading ability without purification and identification of the exact quorum quenching enzymes. Romero et al. found *Tenacibaculum maritimum*—a fish pathogen—could possibly produce acylase targeting C10-HSL [87]. Nithya mentioned a *Bacillus pumilus* isolated from Palk Bay sediments possibly having acylase activity, which could be used for effectively inhibition of QS-regulated virulence expression and biofilm formation of *P. aeruginosa* [88]. Tang et al. screened out 25 strains from flounder with wide AHL-degradation ability, 12 strains of which were indicated to have AHL lactonase activities [33]. Ghani et al. found that a *Labrenzia* sp. isolated from Malaysian seawater could degrade a wide range of AHLs via lactonase activity, yet the quorum quenching enzyme gene was not revealed [89]. Romero et al. screened out 15 marine bacterial strains (belonging to ten genera) that could enzymatically inactivate AHLs (C4-HSL and C12-HSL); some might have lactonase activities while others might have acylase activities [47].

Besides the above studies, some researchers identified the specific genes that encode quorum quenching enzymes. Phelan et al. found that two endospore-forming bacteria (possibly *Bacillus cereus*) from marine sponge have an *aiiA* gene encoding AHL lactonase activity [90]. Kem et al. identified a *bntA* gene and two genes (*mhtA* and *mhtB*) from marine bacteria *Marinobacter* sp. and *Marinobacter nanhaiticus*, respectively. These genes were homologues of acylase gene *pvdQ* in *Pseudomonas aeruginosa* [91]. Gutiérrez-Barranquero et al. found a gene encoding penicillin amidase enzyme (with potential for AHL degradation) in *Paracoccus* sp. from a marine sponge harvested off the west coast of Ireland [92]. Kalia et al. found through genome analysis that three strains belonging to marine gammaproteobacteria possess conserved domains for AHL-lactonases and acylases [93]. Teasdale screened seven strains from various marine environments; six were isolated from marine sediments and belonged to *Bacillus* sp., and one from microbial mats belonged to *Halobacillus* sp. All have the AHL lactonase gene *aiiA* [37]. Rehman et al. isolated seven strains of Proteobacteria from Red Sea sediments, which could degrade AHL molecules of different acyl chain lengths and modifications. Further genome sequencing of three strains discovered AHL lactonase open reading frames (ORFs) mainly belonging to metallo-β-lactamase (MBL) superfamily and AHL acylase ORFs [48]. Interestingly, quorum quenching enzymes were also found in some atypical marine bacteria. Wong et al. isolated a *P. aeruginosa* strain from seawater in Malaysia. The strain had *quiP* and *pvdQ* homologue gene sequences encoding AHL acylase [94,95]. Besides the homologues of the known AHL lactonases and acylases, other enzyme types for QS interference might also exist. Weiland-Bräuer et al. searched for ORFs conferring quorum quenching activity in marine Eukarya-associated bacteria isolates. The ORFs identified in *Photobacterium* sp., *Pseudoalteromonas* sp., and *Vibrio parahaemolyticus* encoded proteins with unknown functions or different functions from the known lactonases or acylases. The functions of these novel proteins remained to be disclosed [45].

Recently, only a few researches provided relatively in-depth and comprehensive studies of quorum quenching enzymes from marine bacteria (Table 2). As reported, AHL lactonases from bacterial origin could be mainly divided into metallo-β-lactamase (MBL) superfamily, α/β hydrolase family, phosphotriesterase (PTE) family, GDSL hydrolase family, etc., based on amino acid sequence features and relationship [96]. However, we found that α/β hydrolase family and PTE family proteins are lacking in marine bacteria-derived lactonases. For marine bacteria-derived acylases, the two listed (Table 2) belong to N-terminal nucleophile (Ntn) hydrolase family. Most of the quorum quenching enzymes have a relatively wide substrate spectrum. Tolerance to heat and wide pH ranges of certain enzymes indicated their promising applications in a wide range of fields.

Since only a limited amount of quorum quenching enzymes has been studied, there is still vast space for researchers to explore novel quorum quenching enzymes from marine bacteria. In several studies, it is assumed that AHL acylase might be more common than lactonase in marine bacteria [29,33,38]. This speculation still needs us to verify with more research results. Besides the known quorum quenching enzyme categories such as AHL lactonase and acylase, we could speculate that other enzymes types in marine bacteria might also participate in AHL degradation, utilization or interference. To determine the existence and exact functioning mechanisms of novel quorum quenching enzymes, biosensor strain assay, HPLC-MS, gene cloning, genome sequencing technologies, etc. should be utilized in combination. To facilitate the application of these enzymes, protein structure analysis and site-directed mutagenesis technologies should be applied to produce structure-modified quorum quenching enzymes with good stability and high efficiency.

## 5. Potential Application Perspectives of QSIs or QSI-Producing Bacteria

The emergence of multidrug-resistant pathogens requires the researchers to find new therapeutic methods. QS interference might become a novel antimicrobial therapy based on non-antibiotic strategy and an attractive alternative to traditional chemical drugs and antibiotics. Therefore, QSIs have aroused increasing attention. As mentioned above, QSIs could either be small molecule chemical compounds or quorum quenching enzymes, both interfering with bacterial QS systems. Either the purified QSIs or probiotic bacteria with QS-inhibitory activities have the potential for applications in various fields, including food preservation, aquaculture, human healthcare, ecological protection, etc., by controlling QS-mediated food spoilage and inhibiting QS-mediated biofilm formation and virulence of pathogens.

To date, applications of marine bacteria-derived QSIs are few and limited. As we summarized in Table 1, marine-derived small molecule QSIs have mainly been used to inhibit biofilm formation and QS-regulated virulence expression in *P. aeruginosa*, *S. aureus*, *Vibrio* sp., and uropathogen *S. marcescens*, to further reduce the toxicity of pathogens. Based on current researches, these small molecule QSIs are promising in curing diseases of human beings and aquatic animals. As for the reported quorum quenching enzymes (Table 2), besides targeting *P. aeruginosa* and *Vibrio* sp., they were used to quench virulence of gastrointestinal pathogens (e.g., *E. coli*), plant pathogen *E. carotovora*, etc., indicating that they might be applied in human healthcare, animal culture, aquaculture and crop cultivation.

A combinational therapy of potential marine bacteria-derived QSIs and traditional antibiotics was also focused. The crude extract of a *Pseudoalteromonas* sp. with QS-inhibitory activity was used in combination with tobramycin against *P. aeruginosa* [43]. Extracts of a marine-derived *Rhizobium* sp. with QS-inhibitory activity were used in conjunction with kanamycin against *P. aeruginosa* [55]. Phenol, 2,4-bis(1,1-dimethylethyl)—a potential small molecule QS inhibitor of marine bacterial origin—was used synergistically with gentamicin against the uropathogen *S. marcescens* [72]. Compared with usage of antibiotics alone, the combination therapy could effectively increase the antimicrobial efficiency of conventional antibiotics. This combined treatment strategy, which might eradicate pathogenic infections more effectively in an early stage, is hopeful to be developed and applied in treatments of various diseases.

Besides the usage of QSIs as therapeutic agents or drugs, production of antibacterial coatings based on natural or modified QSIs seems to be another promising application field. Immobilization of furanone derivatives on polymer materials has been studied to make antibacterial medical devices, which gives us inspirations that marine bacteria-derived QSIs could also be used in medical applications to produce aseptic or antibacterial materials for disease treatments [103].

## 6. Concluding Remarks

The QS interference capabilities of marine bacteria cannot be underestimated, which are realized through QSIs, mainly including small molecules and quorum quenching enzymes. Interestingly, the origins, characteristics, and working mechanisms of QSIs are quite different. For many discovered QSIs, the latter two aspects are still unsolved mysteries. Due to the microbial diversity in the marine environment and the antimicrobial activities of QSIs, marine bacteria with QS-inhibitory activities and potential QSIs remain to be explored. In the future studies, besides large-scale screening of QS-inhibitory bacteria and discovery of QSIs, QS antagonistic mechanisms should be more effectively targeted and elucidated using combined methods and technologies, especially those at cellular and molecular levels. Also, to realize the practical applications of QSIs, their toxic effects against human beings, animals or plants should be assessed more comprehensively with multiple approaches. Only with absolute security, QSIs could truly realize their value and be put into production of antimicrobial agents or materials for disease prevention and treatments.

## Figures and Tables

**Table 1:** Small molecule QS inhibitors. Structures, origin, working concentrations, mechanisms and effects.

Structures of QS Inhibitors	Marine Bacterial Origins	Sources of the Bacteria	QS-Inhibitory Concentration	Anti-QS Working Mechanisms	Effects	References
**Cyclic peptides and a linear peptide**						
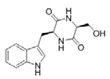 Cyclo (Trp-Ser)	*Rheinheimera aquimaris*	Marine sediment surrounding the Yellow Sea in Qingdao, China	sub-MIC: 0.2 mg/mL	Probably interfere in the stability of LasR receptor	Suppress biofilm formation and QS-regulated pyocyanin and elastase activity in *Pseudomonas aeruginosa*	[59]
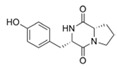 Cyclo-l-proline-l-tyrosine	*Bacillus cereus*	Marine sediment along the Rhode Island coastline	-	Possibly inhibit *Vibrio* sp. LuxR	-	[37]
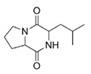 Cyclo (l-leucyl-l-prolyl)	*Bacillus amyloliquefaciens*	Mangrove rhizosphere of Palk Strait, Bay of Bengal, India	sub-MIC: 100 μg/mL	-	Inhibit QS-controlled biofilm and virulence factors (prodigiosin, extracellular polymeric substance, protease, and lipase) production in uropathogen *Serratia marcescens*	[60,61]
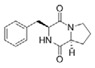 Cyclo (l-Pro-l-Phe)	*Marinobacter* sp.	A hypersaline cyanobacterial mat from wadi Muqshin in Oman, off the Arabian Sea coast	sub-MIC: μM grade	Possibly compete with signal molecules AHLs and inhibit QS	-	[53]
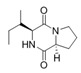 Cyclo (l-Pro-l-isoLeu)						
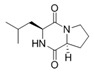 Cyclo (l-Pro-lLeu)						
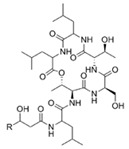 Ngercheumicin F (R = C11H21), Ngercheumicin G (R = C11H23), Ngercheumicin H (R = C13H25), Ngercheumicin I (R = C13H27) (Cyclodepsipeptides)	*Photobacterium halotolerans*	Mussel surface in the tropical Pacific	20 μg/mL	Interfere with *agr* QS	Reduce expression of virulence genes of *hla* (hemolysin) and *rnaIII* (an effector molecule) of *agr* QS in *Staphylococcus aureus*	[62]
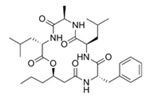 Solonamide A (Fatty acid-l-Phe- d-Leu-d-Ala-l-Leu)	*Photobacterium halotolerans*	Mussel surface in the tropical Pacific	μg/mL–mg/mL	Possibly interfere with the *agr* QS system by competing with AIP for binding to sensor histidine kinase AgrC	Inhibit virulence gene expression of *hla* (hemolysin), *rnaIII* (an effector molecule of *agr*) and *psm*α (phenol soluble modulins) in *Staphylococcus aureus*, and reduce toxicity of *S. aureus* toward human neutrophils	[63,64]
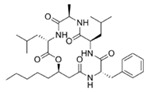 Solonamide B (Fatty acid-l-Phe- d-Leu-d-Ala-l-Leu)						
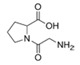 Linear dipeptide proline-glycine	*Streptomyces* sp.	Marine invertebrates from the western coast of India	sub-MIC: 0.1 mg/mL	-	Inhibit QS-mediated virulence factors (swarming, pyocyanin pigmentation, biofilm formation, rhamnolipid production, and Las A) in *Pseudomonas aeruginosa*	[32]
**Amides**						
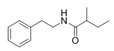 2-Methyl-*N*-(2′-phenylethyl) butyramide	*Oceanobacillus* sp.	Marine environment	sub-MIC: μg/mL grade	-	Inhibit pyocyanin production, elastase activity and biofilm formation of *Pseudomonas aeruginosa*	[65]
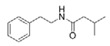 3-Methyl-*N*-(2′-phenylethyl)-butyramide						
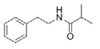 *N*-(2′-Phenylethyl)-isobutyramide	*Halobacillus salinus*	Sea grass sample from Point Judith Salt Pond, Rhode Island	sub-MIC: μg/mL grade	Possibly compete with AHLs for receptor binding	-	[66]
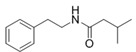 3-Methyl-*N*-(2′-phenylethyl)-butyramide						
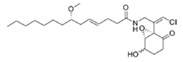 8-*epi*-Malyngamide C (8R) and malyngamide C (8S)	cyanobacterium *Lyngbya majuscula*	Off Bush Key, Florida	μM grade	-	-	[67]
Fatty Acids and phenol derivatives						
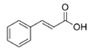 cinnamic acid	*Streptomyces* sp.	Marine invertebrates from the western coast of India	sub-MIC: 0.1 mg/mL	-	Inhibit QS-mediated virulence factors (swarming, biofilm formation, LasA, pyocyanin and rhamnolipid production) in *Pseudomonas aeruginosa*	[32]
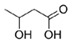 β-Hydroxy butyric acid (degradation product of PHB)	*Brevibacterium casei* (sources of PHB)	Marine sponge *Dendrilla nigra*	50 μg/mL (PHB)	Possibly by AHL degradation	Control biofilm formation, colonization capacity, motility and hemolysin activity of *Vibrio* sp.	[68]
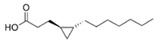 Lyngbyoic acid	cyanobacterium *Lyngbya cf. majuscula*	Indian River Lagoon near Fort Pierce, Florida	μM–mM grade	Possibly inhibit *lasR* signaling by competing with AHL for binding LasR	Reduce pyocyanin and elastase (LasB) in *Pseudomonas aeruginosa*	[69]
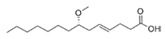 Lyngbic acid	cyanobacterium	Corals from the Florida Keys and Belize	nM–μM grade	Compete with CAI-1 for binding to QS signal receptor CqsS	-	[70]
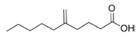 Pitinoic acid A	cyanobacterium similar to *Lyngbya* sp.	A channel at the north end of Piti Bay at Guam	sub-MIC: μM–mM grade	-	Inhibit expression of QS-related virulence factor LasB (elastase) and the pyocyanin in *Pseudomonas aeruginosa*	[71]
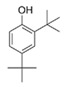 Phenol, 2,4-bis (1,1-dimethylethyl)	*Vibrio alginolyticus*	Red seaweed *Gracilaria gracilis* from the Karankadu coastal region of Palk Bay, India	sub-MIC: μg/mL grade	-	Inhibit QS-mediated biofilm formation and virulence factor production (protease, hemolysin, lipase, prodigiosin, and extracellular polysaccharide) in the uropathogen *Serratia marcescens*	[72]
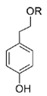 Tyrosol/tyrosol acetate (R = H or Ac)	*Oceanobacillus profundus*	Caribbean soft coral *Antillogorgia elisabethae*	-	-	-	[49]
**AHL analogs**						
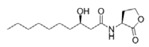 *N*-(3-Hydroxydecanoyl)-l-homoserine lactone	*Phaeobacter inhibens*	Inner surface of an oyster shell	μM grade	Possibly by competitive inhibition of AHL-mediated QS	Inhibit virulence factor metalloprotease in *Vbrio coralliilyticus*	[73]
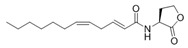 *N*-(Dodecanoyl-2,5-diene)-l-homoserine lactone						
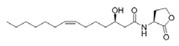 *O*-(3-Hydroxytetradecanoyl-7-ene)-l-homoserine lactone						
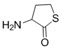 d,l-Homocysteine thiolactone	*Staphylococcus hominis*	coral species (*Pocillopora damicornis*) in Xishan Islands, South China Sea	above 0.0625 μg/mL	Possibly compete with AHL in occupying the AHL receptor	Suppress biofilm formation and elastase production in *Pseudomonas aeruginosa*	[31]
**Others**						
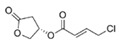 Honaucin A	cyanobacterium *Leptolyngbya crossbyana*	Corals on the Hawaiian coast	μM grade	Possibly compete with AHL in occupying the AHL receptor	-	[74]
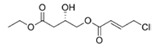 Honaucin B				-		
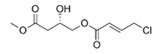 Honaucin C				-		
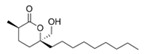 Malyngolide	Cyanobacterium *Lyngbya majuscula*	Indian River Lagoon, USA	sub-MIC: 3.57–57 μM	Possibly inhibit QS by reducing or partially blocking the expression of *lasR*	Inhibit Las QS-dependent production of elastase by *Pseudomonas aeruginosa*, and possibly help the cyanobacterium to control growth of heterotrophic bacteria	[75]
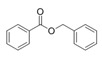 Benzyl benzoate	*Oceanobacillus* sp.	Marine environment	sub-MIC: μg/mL grade	-	Inhibit pyocyanin production, elastase activity and biofilm formation of *Pseudomonas aeruginosa*	[65]
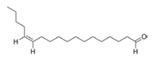 13Z-Octadecenal	*Streptomyces griseoincarnatus*	Marine sponge *Callyspongia* sp. from Gulf of Mannar, India	-	Bind to AHL synthase LasI of *Pseudomonas aeruginosa*	Possibly inhibit *Pseudomonas aeruginosa* biofilm	[76]

Note: MIC: minimum inhibitory concentration for microorganism growth.

**Table 2:** Marine bacteria derived-quorum quenching enzymes. Categories, origins, substrate spectrums, environmental tolerance and applications.

Quorum Quenching Enzymes	Protein Sequence Accession Number	Protein Superfamily/Family	Bacteria	Marine Origin of the Bacteria	Substrate Spectrum	Environmental Tolerance	Applications	References
AHL lactonase Aii20J	AKN24544	Metallo-β-lactamase	*Tenacibaculum* sp.	Sediment of fish culture tank, Spain	AHLs (C4-HSL, C6-HSL, C8-HSL, C10-HSL, C12-HSL, C14-HSL, OC6-HSL, OC10-HSL, OC12-HSL, OC13-HSL, OC14-HSL, OHC10-HSL, OHC12-HSL)	With heat resistance in cell extracts, tolerance to protease and wide pH range 3–9	Quench AHL-mediated acid resistance in *Escherichia coli* (gastrointestinal pathogens)	[97]
AHL lactonase AiiA	CAJ84442	Metallo-β-lactamase	*Bacillus cereus*	Seawater samples of South China Sea	OC8-HSL	-	-	[98]
AHL lactonase MomL	AIY30473	Metallo-β-lactamase	*Muricauda olearia*	Skin mucus of flounders from marine fish farms in China	AHLs (C4-HSL, C6-HSL, C8-HSL, OC6-HSL, OC8-HSL, OC10-HSL)	No heat resistance, tolerance to pH range 7–11	Attenuate the virulence (extracellular protease activity and pyocyanin production) of *Pseudomonas aeruginosa*, increase the survival of *Caenorhabditis elegans*	[99]
AHL lactonase QsdH	(Included in) AFV15299	GDSL hydrolase	*Pseudoalteromonas byunsanensis*	Marine Culture Collection of China	AHL (C4HSL, C6HSL, C8HSL, C10HSL, C12HSL, C14HSL, OC6-HSL, OC8-HSL)	No heat resistance	Attenuate the plant pathogenicity of *Erwinia carotovora*	[100]
AHL lactonase RmmL	AYM45058	Metallo-β-lactamase	*Ruegeria mobilis*	Healthy shrimp larvae	AHL (C6-HSL, C8-HSL, C10-HSL, C12-HSL, OC6-HSL, OC8-HSL, OC10-HSL, OC12-HSL, OC14-HSL)	No heat resistance, tolerance to pH range 2–9	Reduce the production of virulent factor pyocyanin by *Pseudomonas aeruginosa*	[96]
AHL lactonase FiaL	-	Metallo-β-lactamase	*Flaviramulus ichthyoenteri*	Intestine of cultured healthy flounder in China	AHL (C6-HSL, C8-HSL, C10-HSL, C12-HSL, C14-HSL, OC6-HSL, OC8-HSL, OC10-HSL, OC12-HSL, OC14-HSL)	-	-	[101]
AHL acylase MhtA	ENO13542	Ntn-hydrolases	*Marinobacter nanhaiticus*	Sediment of the South China Sea	AHL (C12-HSL)	-	-	[91]
AHL acylase PfmA	ASS36259	Ntn hydrolase	*Pseudoalteromonas flavipulchra*	Water used to rear healthy turbot in China	AHL (C10-HSL, C12-HSL, C14-HSL, OC12-HSL, OC14-HSL, OHC14-HSL)	No heat resistance, tolerance to pH range 5–11	Reduce virulence factor protease production in *Vibrio anguillarum*, reduce protease and pyocyanin in *Pseudomonas aeruginosa*, increase survival of infected *Artemia*	[102]

Note: no heat resistance means that the enzyme activity drops at temperature higher than 50 °C.
